# Exploring the five different genes associated with PKCα in bladder cancer based on gene expression microarray

**DOI:** 10.1111/jcmm.16284

**Published:** 2021-01-15

**Authors:** Xiaotong Zhang, Jiarun Zhang, Hao Zhang, Yang Liu, Lei Yin, Xi Liu, Xuejie Li, Xiuyue Yu, Jinlong Yao, Zhe Zhang, Chuize Kong

**Affiliations:** ^1^ Department of Urology The First Hospital of China Medical University Shenyang China

**Keywords:** apoptosis, bladder cancer, microarray, NF‐kb, PKCα, proliferation

## Abstract

Much progress has been made in understanding the mechanism of bladder cancer (BC) progression. Protein kinase C‐α (PKCα) is overexpressed in many kinds of cancers. Additionally, PKCα is considered an oncogene that regulates proliferation, invasion, migration, apoptosis and cell cycle in multiple cancers. However, the mechanism underlying how these cellular processes are regulated by PKCα remains unknown. In the present study, we used PKCα siRNA to knock down PKCα gene expression and found that down‐regulation of PKCα could significantly inhibit cell proliferation, migration and invasion and induce apoptosis and G1/S cell cycle arrest in vitro. Overexpression of PKCα promotes tumour growth in vivo. We applied cDNA microarray technology to detect the differential gene expression in J82 cells with PKCα knockdown and found that five key genes (BIRC2, BIRC3, CDK4, TRAF1 and BMP4) were involved in proliferation and apoptosis according to GO analysis and pathway analyses. Correlation analysis revealed a moderate positive correlation between PKCα expression and the expression of five downstream genes. BIRC2 and BIRC3 inhibit apoptosis, whereas CDK4, TRAF1 and BMP4 promote proliferation. Essentially, all five of these target genes participated in proliferation, and apoptosis was regulated by PKCα via the NF‐kB signalling pathway.

## 1 INTRODUCTION

Bladder cancer (BC) is the ninth most common cancer worldwide. The incidence of BC in men is approximately three times that in women, as BC accounts for 7% of new cancer cases in men and 2% in women.^1^ BC is the fourth most common cancer in men after prostate cancer, lung cancer and colorectal cancer. Haematuria is a presenting symptom in 85%‐90% of patients with BC. Approximately 98% of all BCs are epithelial malignancies; approximately 90% are transitional cell carcinomas (TCCs), and the remaining 8% are mainly adenocarcinomas, squamous cell carcinomas or undifferentiated carcinomas. Approximately 15% of patients with BC are found to have regional or distant metastases, approximately 30%‐40% of patients with invasive disease develop distant metastases despite radical cystectomy or definitive radiotherapy, and approximately 70% of patients exhibit recurrence after transurethral resection of bladder tumour (TURBT).^2^, ^3^ Therefore, it is critical to identify highly sensitive diagnostic and prognostic markers for the treatment of this disease.

Protein kinase C‐α (PKCα), also named PRKCA, is a member of the protein kinase C family. Accumulating evidence has demonstrated that PKCα is overexpressed in many kinds of cancers, such as breast, lung, renal, and colorectal cancers.^4^, ^5^, ^6^ In addition, PKCα is considered to be an oncogene and regulates the proliferation, invasion, migration and apoptosis of tumour cells via activation of the mTOR‐signalling pathway.^7^ In addition to its tumorigenic activity, PKCα is involved in diverse functions in cell development and has been implicated in many pathological processes, such as inflammation, oxidative stress, myelodysplastic syndromes and diabetic nephropathy.^8^

Our previous study demonstrated that PKCα played a significant role in tumorigenesis of BC. In the present study, we aimed to identify the pathogenic mechanisms underlying the antineoplastic effects induced by PKCα knockdown. First, we employed cDNA microarray technology to detect the expression of target genes on a genome‐wide level using BC cells with PKCα knockdown. Using GO and KEGG enrichment analyses, we found that the expression of all five target genes was positively correlated with PKCα expression and demonstrated that all five target genes were regulated by PKCα via the NF‐kB signalling pathway. All these findings contribute to our knowledge regarding BC progression and to the development of a novel therapy for BC.

## 2 MATERIALS AND METHODS

### 2.1 Clinical samples and animal studies

A total of 20 BC samples and 20 adjacent normal tissues were collected from patients who underwent resection between 2016 and 2017 in the First Hospital of China Medical University in China. Adjacent normal tissues were collected at a distance of more than 5 cm from tumour. All tissues, including the tumour tissues and adjacent normal tissues, were processed for histological examination. The patients’ clinical characteristics are listed in Table 1. Tumour formation experiments in nude mice (BALB/C‐nu/nu, female, 4 weeks old) were performed at the Experimental Animal Center of China Medical University. All mice were housed and maintained under specific pathogen‐free conditions. The clinical samples and animal studies were approved by Medical Ethics Committee and Animal Welfare and Ethical Committee, respectively.

**TABLE 1:** Correlations between the proportion of PKCα and clinicopathological features in 20 BC patients

Characteristics	Case	PKCα expression		*P* value
		Low	High	
All cases	20	12	8	
Age (years)				
<65	8	4	4	.4561
≥65	12	8	4	
Gender				
Male	15	8	7	.2918
Female	5	4	1	
TNM stage				
pTa‐pT1	6	3	3	.5501
pT2‐pT4	14	9	5	
Histological grade				
Low	8	6	2	.2636
High	12	6	6	
Tumour size(cm)				
<3 cm	11	7	4	.7136
≥3 cm	9	5	4	

### 2.2 Cell culture and transfection

SV‐HUC‐1, which was considered to be the immortalized normal urothelial cell line, and BC cell lines J82, T24 and UMUC3 were obtained from Chinese Academy of Sciences Cell Bank (CASCB, China). All cells were cultured in RPMI 1640 medium (Gibco, USA) containing 10% heat‐inactivated foetal bovine serum (FBS) (Gibco) and at 37°C in 5% CO_2_.

Small interfering RNAs (siRNAs) targeting PKCα, BIRC2, BIRC3, CDK4, TRAF1 and BMP4, and the PKCα overexpression plasmid were synthesized by JTSBIO Co. Lipofectamine 3000 Reagent (Life Technologies Corporation, USA) was used for transfection.

### 2.3 Quantitative RT‐PCR (qRT‐PCR)

Total RNA was extracted using an miRcute miRNA Isolation Kit (TIANGEN, Beijing) in accordance with the manufacturer's instructions. For RT‐PCR, 1500 ng of extracted RNA was directly reverse‐transcribed using Prime Script RT Master Mix (Takara, Dalian) and random primers. To quantify the amounts of mRNA, real‐time PCR analyses were quantified by SYBR^®^ Premix Ex Taq™ Kit (Takara). GAPDH expression was considered to be internal control. All analyses were performed using a Thermal Cycler Dice™ Real‐Time TP800 System (Takara, Kyoto, Japan). The ΔΔCT method was employed to calculate the relative expression of different genes. The primers used are listed in Table 2.

**TABLE 2:** Real‐time PCR primer sequences

Primer name	Primer sequences
PKCα forward	5′‐ATGTCACAGTACGAGATGCAAAA‐3′
PKCα reverse	5′‐GCTTTCATTCTTGGGATCAGGAA‐3′
GAPDH forward	5′‐AGAAGGCTGGGGCTCATTTG‐3′
GAPDH reverse	5′‐AGGGGCCATCCACAGTCTTC‐3′
BIRC2 forward	5′‐AGCACGATCTTGTCAGATTGG‐3′
BIRC2 reverse	5′‐GGCGGGGAAAGTTGAATATGTA‐3′
BIRC3 forward	5′‐TTTCCGTGGCTCTTATTCAAACT‐3′
BIRC3 reverse	5′‐GCACAGTGGTAGGAACTTCTCAT‐3′
CDK4 forward	5′‐CTGGTGTTTGAGCATGTAGACC‐3′
CDK4 reverse	5′‐GATCCTTGATCGTTTCGGCTG‐3′
TRAF1 forward	5′‐TCCTGTGGAAGATCACCAATGT‐3′
TRAF1 reverse	5′‐GCAGGCACAACTTGTAGCC‐3′
BMP4 forward	5′‐ATGATTCCTGGTAACCGAATGC‐3′
BMP4 reverse	5′‐CCCCGTCTCAGGTATCAAACT‐3′

### 2.4 Western blot analysis

Total protein was extracted using RIPA buffer, and 30 µg of protein was separated by sodium dodecyl sulphate‐polyacrylamide gel electrophoresis (SDS‐PAGE) on a 10% gel. Then, proteins were transferred onto polyvinylidene fluoride (PVDF) membranes. The membranes were blocked at room temperature for 1 hour. Then, all membranes were incubated with anti‐PKCα (1:1000, ab32376; Abcam, MA, USA), anti‐BIRC2 (1:1000, ab108361; Abcam), anti‐BIRC3 (1:1000, ab32059; Abcam), anti‐CDK4 (1:1000, ab108357; Abcam), anti‐TRAF1 (1:1000, ab197684; Abcam), anti‐BMP4 (1:1000, ab124715; Abcam), anti‐p‐IkB (1:1000, ab92700; Abcam), anti‐Bcl2 (1:1000, ab32124; Abcam) or anti‐GAPDH (1:5000; Santa Cruz, CA, USA) primary antibodies. After the membranes were incubated with secondary antibodies, they were washed three times with Tris‐buffered saline/Tween‐20 (TBST). A chemiluminescence system (Bio‐Rad, CA, USA) was used to detect the immunoreactive signals.

### 2.5 RTCA (real‐time cell assay)

Transfected cells were washed with phosphate‐buffered saline (PBS) and dissociated with trypsin. Then, the cells were planted in cell culture E‐plates of 3500 cells per well and cultured in a humidified incubator. Cell growth curves were automatically recorded in real time with an xCELLigence System (Roche Applied Sciences), and the cell index was monitored for 3 days.

### 2.6 EdU (5‐ethynyl‐2'‐deoxyuridine) assay

A total of 5 × 10^3^ cells/well cells were put into 96‑well plates after cell transfection. Then, a concentration 50 µmol/L of EdU (Beyotime Co., Shanghai, China) was added into 96‑well plates for 4 hour at 37°C in 5% CO_2_ incubator. Cells were treated with 4% formaldehyde for 15 minutes and 0.3% Triton X‐100 for 15 minutes at room temperature. Then, all cells were treated following the manufacturer's instructions. Finally, cells were visualized under a fluorescent microscope (magnification, ×400).

### 2.7 Transwell assay

Transwell assays were used to assess the invasion and migration of BC cells by using or without using Matrigel, respectively. In all, 2 × 10^4^ BC cells (T24 and UMUC3) were suspended in 200 µL serum‐free RPMI 1640 medium and incubated in incubator. After 24 hour, the non‐invading or non‐migratory cells were removed with a cotton tip, and the remaining cells on the bottom were stained with 0.1% crystal violet.

### 2.8 Flow cytometry assay

BC cell apoptosis was detected by flow cytometry following the manufacturer's instructions. Cells evaluated for apoptosis were incubated in the dark with 5 μL Annexin V‐FITC and 5 μL propidium iodide (PI). Cells evaluated for the cell cycle were incubated in the dark with propidium iodide (20 mg/mL). All cells were then analysed by flow cytometry (BD Biosciences, Franklin Lakes, NJ) on a FACScan instrument (Becton Dickinson, NY).

### 2.9 Immunohistochemistry (IHC) analysis

Paraffin‐embedded tissues from nude mice were cut into 4‐μm slides. Rabbit anti‐Ki67 antibodies were purchased from Cell Signaling Technology Company. IHC analysis was used in accordance with the procedure. Images were obtained at 200× or 400× magnification.

### 2.10 Statistical analysis

All data are expressed as the mean ± SD for three independent experiments: *P < 0.05, **P < 0.01 and ***P < 0.001. Data analyses were carried out using GraphPad Prism 7.0 (GraphPad Software, La Jolla, CA, USA) and SPSS software ver. 20.0 (SPSS, Inc, Chicago, IL, USA). Probability (*P*) values < 0.05, as calculated using Student's t test as appropriate, were considered statistically significant. The expression of clinical samples data detected by RT‐PCR, presented as Mann‐Whitney U test was used for significant analysis.

## 3 RESULTS

### 3.1 PKCα expression is up‐regulated and exerts an oncogenic role in bladder cancer in vitro

**FIGURE 1:**
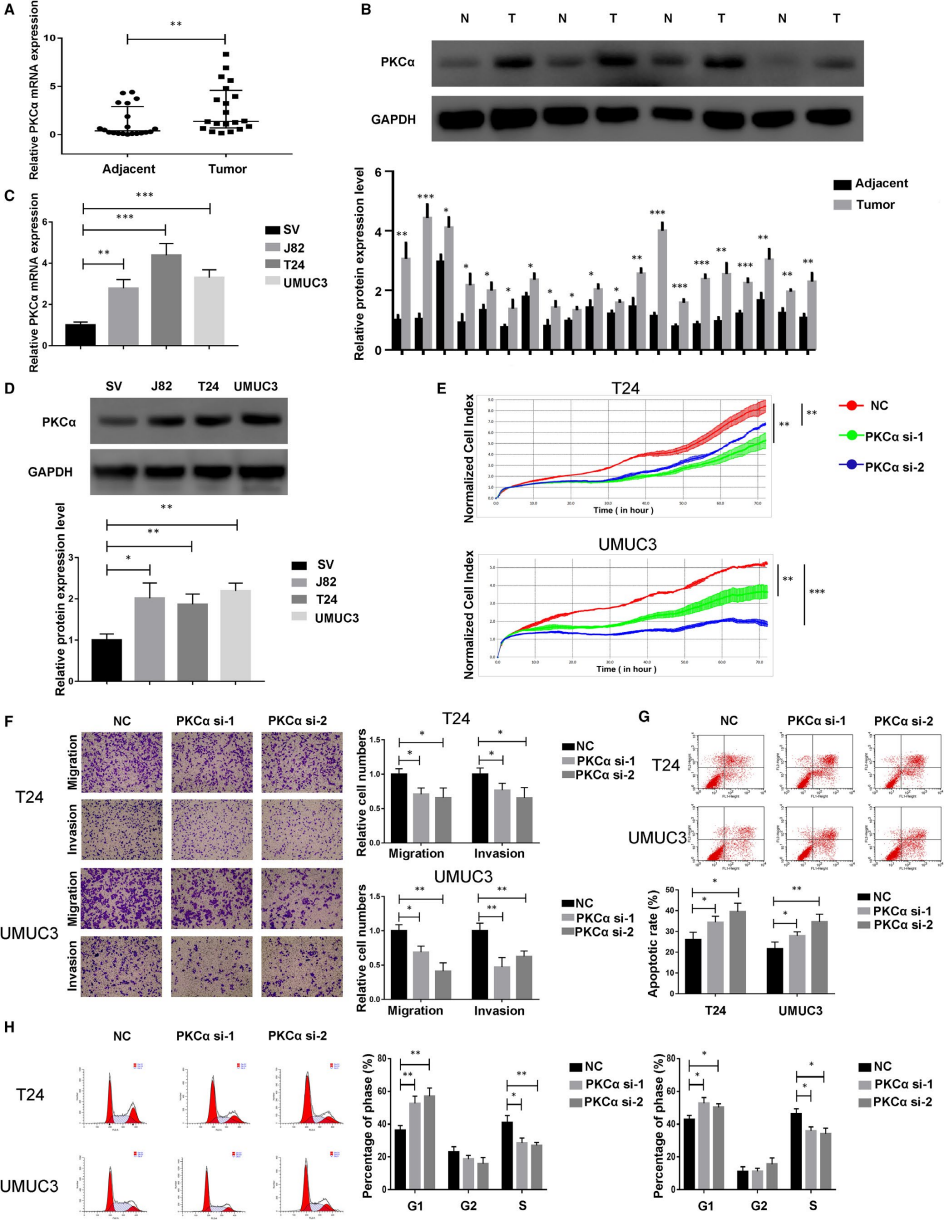
PKCα expression is up‐regulated and exerts an oncogenic role in bladder cancer in vitro. (A and B) PKCα expression in bladder cancer tissues (T) and matched adjacent normal tissues (N) according to qRT‐PCR (n = 40) and Western blotting (n = 40). (C and D) PKCα expression in three bladder cancer cell lines and SV‐HUC‐1 (SV) cells according to qRT‐PCR and Western blotting. (E) T24 and UMUC3 cells were transfected with PKCα siRNA, and proliferation was detected by the RTCA. (F) Cell migration and invasion in treated T24 and UMUC3 cells were performed with transwell assays with and without Matrigel, respectively. (G) Apoptosis was estimated in T24 and UMUC3 cells using flow cytometry. (H) Cell cycle was detected in T24 and UMUC3 cells using flow cytometry

As accumulating evidence has proven that PKCα is overexpressed in multiple cancers, we first re‐confirmed whether PKCα expression was up‐regulated in BC. We collected 20 pairs of BC tissues and normal adjacent tissues and analysed them by qRT‐PCR, and analysed 20 pairs of BC tissues and normal adjacent tissues by Western blotting. The results showed that PKCα was overexpressed in BC tissues (Figure 1A‐B). In addition, we found that PKCα showed high expression in 3 BC cell lines (J82, T24, UMUC3) compared with that in SV‐HUC‐1 (SV) cells, which are considered an immortalized normal urothelial cell line (Figure 1C‐D).

Depending on the previous results, PKCα was overexpressed in BC tissues and cells compared with normal tissues and SV cells, respectively, which prompted us to investigate the function of PKCα on cell survival activities. We first used PKCα siRNA (PKCα si) to determine the role of PKCα on the proliferative ability of BC cells, and the results showed that PKCα down‐regulation could significantly inhibit cell proliferation (Figure 1E). Meanwhile, the functions of PKCα in regulating migration and invasion were also evaluated by transwell migration and invasion assays, which indicated that PKCα down‐regulation could also inhibit cell migration and invasion (Figure 1F). Additionally, apoptosis and cell cycle experiments assessed by flow cytometry also showed that PKCα siRNA could induce apoptosis and G1/S cell cycle arrest in T24 and UMUC3 cells (Figure 1G‐H).

### 3.2 PKCα plays an oncogenic role in bladder cancer in vivo

**FIGURE 2:**
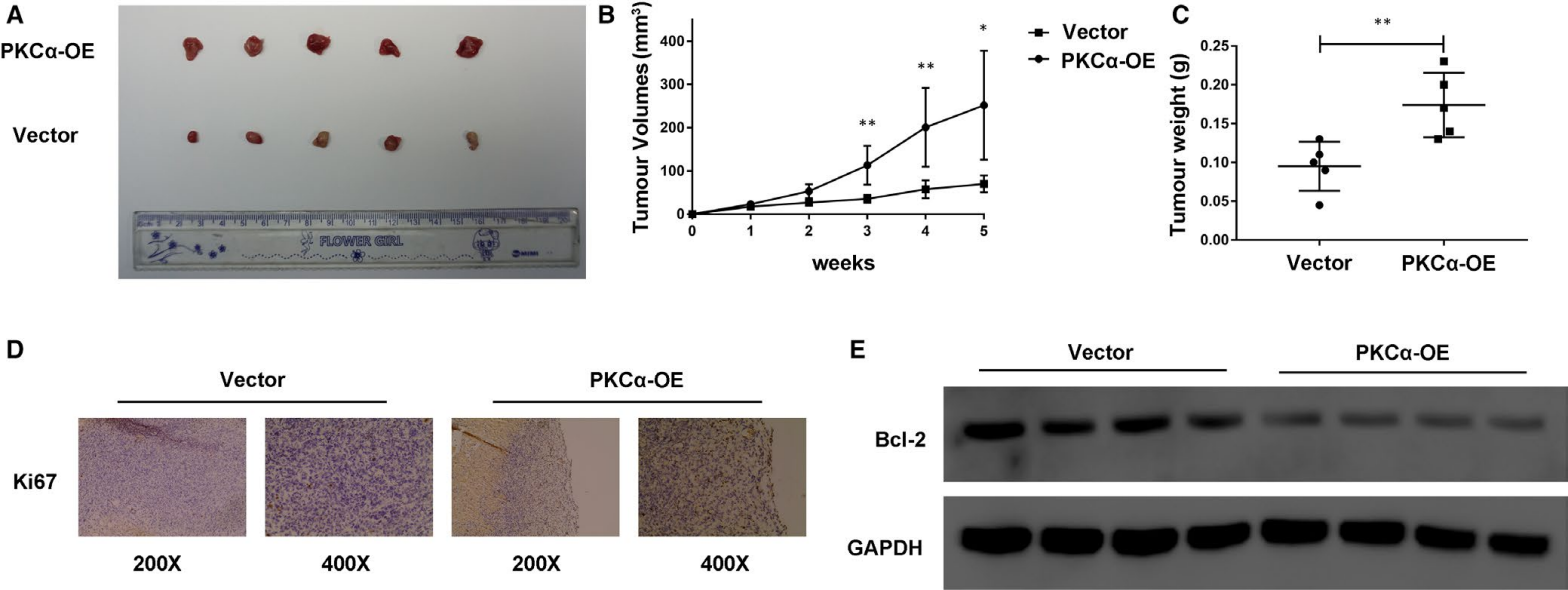
PKCα plays an oncogenic role in bladder cancer in vivo. (A) Representative images of stably transfected UMUC3 cell–derived and xenografted tumours in BALB/c nude mice. (B) The tumour volumes of the two groups were measured every week, and growth curves were presented. (C) The tumour weights of the two groups were measured using electronic scales. (D) The expression of Ki‐67 in xenograft tumours was measured using IHC. (E) The relative expression of Bcl‐2 in xenograft tumours from the two groups was measured using Western blot

As demonstrated by the oncogenic role of PKCα in BC, we next decided to investigate whether PKCα overexpression affects tumour growth in vivo. A total of 1.5 × 107 UMUC3 cells transfected with PKCα‐overexpressing plasmid (PKCα‐OE) and PKCα‐vector (Vector) were subcutaneously injected into BALB/c nude mice (two groups, n = 5 per group). After the injections, the tumour sizes and weights were measured, and we observed that all the tumours in the PKCα‐overexpressing plasmid group increased in size and weight compared with those in the PKCα‐vector group (Figure 2A‐C). In addition, Ki‐67 expression was qualitatively measured by IHC analysis, and Bcl‐2 expression was decreased in the PKCα‐overexpressing plasmid group as assessed by Western blot (Figure 2D‐E). In conclusion, these results prove that PKCα could efficiently promote tumour formation of BC in vivo.

### 3.3 Gene expression microarray analyses of PKCα target genes in bladder cancer cells

**FIGURE 3:**
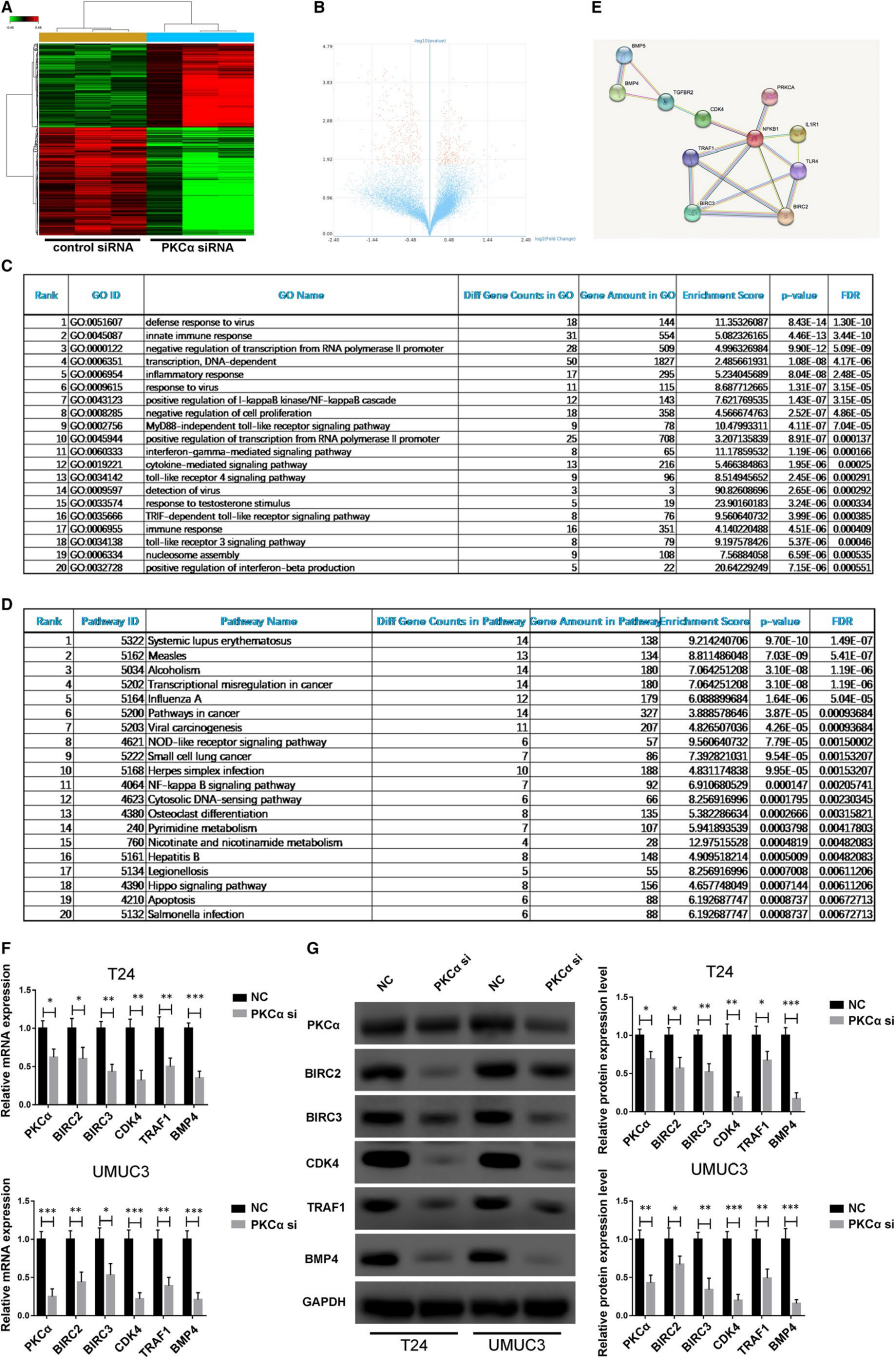
Gene expression microarray analysis of PKCα target genes in bladder cancer cells. (A and B) Representative heat map and volcano maps showing the expression of different target genes in the normal control group and the PKCα siRNA group. (C and D) The top 20 affected biological processes or pathways in PKCα knockdown J82 cells based on GO and KEGG pathway analyses. (E) Schematic of the protein‐protein interaction analysis using STRING software. (F and G) Relative expression of the five key genes in T24 and UMUC3 cells with PKCα knockdown was detected with qRT‐PCR and Western blotting

Having confirmed that PKCα participated in cellular function in BC cells, we next explored the mechanisms underlying how PKCα regulates cell function. We used cDNA microarray technology to detect target gene expression levels on a genome‐wide scale using BC cells with PKCα knockdown. As a result, 500 genes were identified as being differentially expressed (Q < 0.05, *P* < 0.05, differential gene = 500) in PKCα knockdown cells (Figure 3A‐B). GO analysis and pathway analysis were performed with the target genes of PKCα, and biological processes and molecular functions for GO analysis, and signalling pathways for pathway analysis were associated with PKCα knockdown. According to the up‐regulated and down‐regulated transcripts, a total of 80 GO terms and 46 pathways were eventually obtained. The main analysis for the top 20 GO terms was defence response to virus, innate immune response and negative regulation of transcription from RNA polymerase II promoter. KEGG pathway analysis for the top 20 pathways revealed systemic lupus erythematosus, measles, alcoholism and transcriptional misregulation in cancer (Figure 3C‐D). In addition, cellular proliferation, apoptosis processes and the NF‐kB signalling pathway were also correlated with PKCα. Using STRING software, protein‐protein interaction analysis was performed to screen for enriched candidate genes regulated by PKCα in BC cells (Figure 3E). Finally, we identified five key genes (BIRC2, BIRC3, CDK4, TRAF1 and BMP4) regulated by PKCα and confirmed that they are regulated by PKCα by using PKCα siRNA in T24 and UMUC3 cells and performing qRT‐PCR and Western blotting (Figure 3F‐G).

### 3.4 Analysis of the correlation between the identified key genes and PKCα in bladder cancer tissues

**FIGURE 4:**
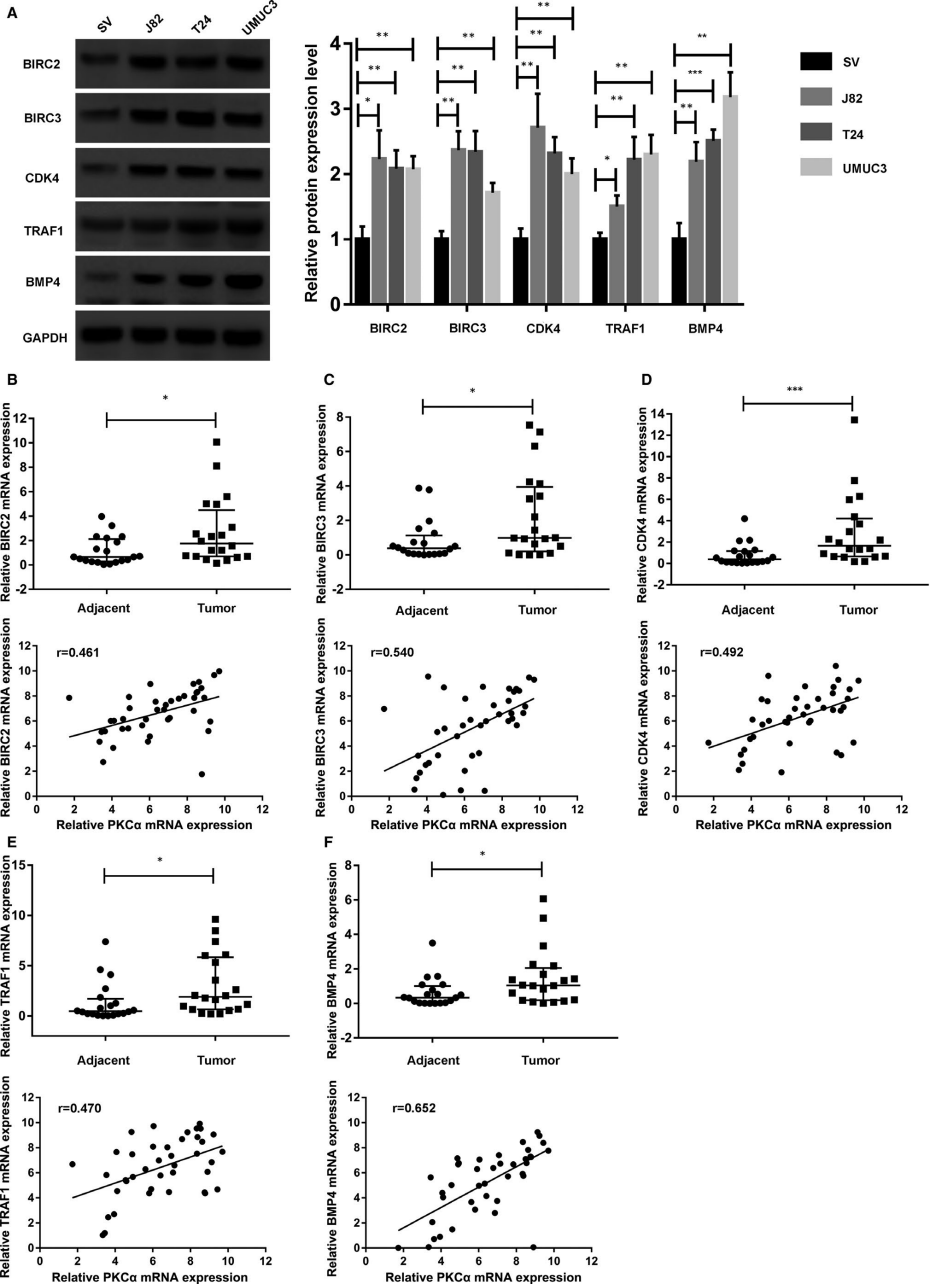
Analysis of the correlation between the five key genes and PKCα in bladder cancer tissues. (A) Five target genes (BIRC2, BIRC3, CDK4, TRAF1 and BMP4) expression in J82, T24 and UMUC3 bladder cancer cell lines and SV‐HUC‐1 cells according to Western blotting. (B, C, D, E and F) Five target genes (BIRC2, BIRC3, CDK4, TRAF1 and BMP4) expression in bladder cancer tissues and matched adjacent normal tissues according to qRT‐PCR (n = 40). A moderate positive correlation between the expression of PKCα and that of the five target genes (BIRC2, BIRC3, CDK4, TRAF1 and BMP4) was identified base on Pearson's correlation analysis

To explore the role of five key genes in BC, we first assessed the expression of these genes in bladder cancer cells, and the Western blotting results showed that all five genes were overexpressed in J82, T24 and UMUC3 cells compared with that in SV‐HUC‐1 cells (Figure 4A). Then, a total of 20 pairs of BC tissues and normal adjacent tissues were analysed by qRT‐PCR to measure the five key genes expression, and all five downstream genes were significantly up‐regulated in the BC tissues. Correlation analysis also revealed a moderate positive correlation between the PKCα expression and each of the five downstream genes (BIRC2, BIRC3, CDK4, TRAF1 and BMP4) (Figure 4B‐F).

### 3.5 PKCα affects cell proliferation and apoptosis in an NF‐kB–dependent manner in bladder cancer cells

**FIGURE 5:**
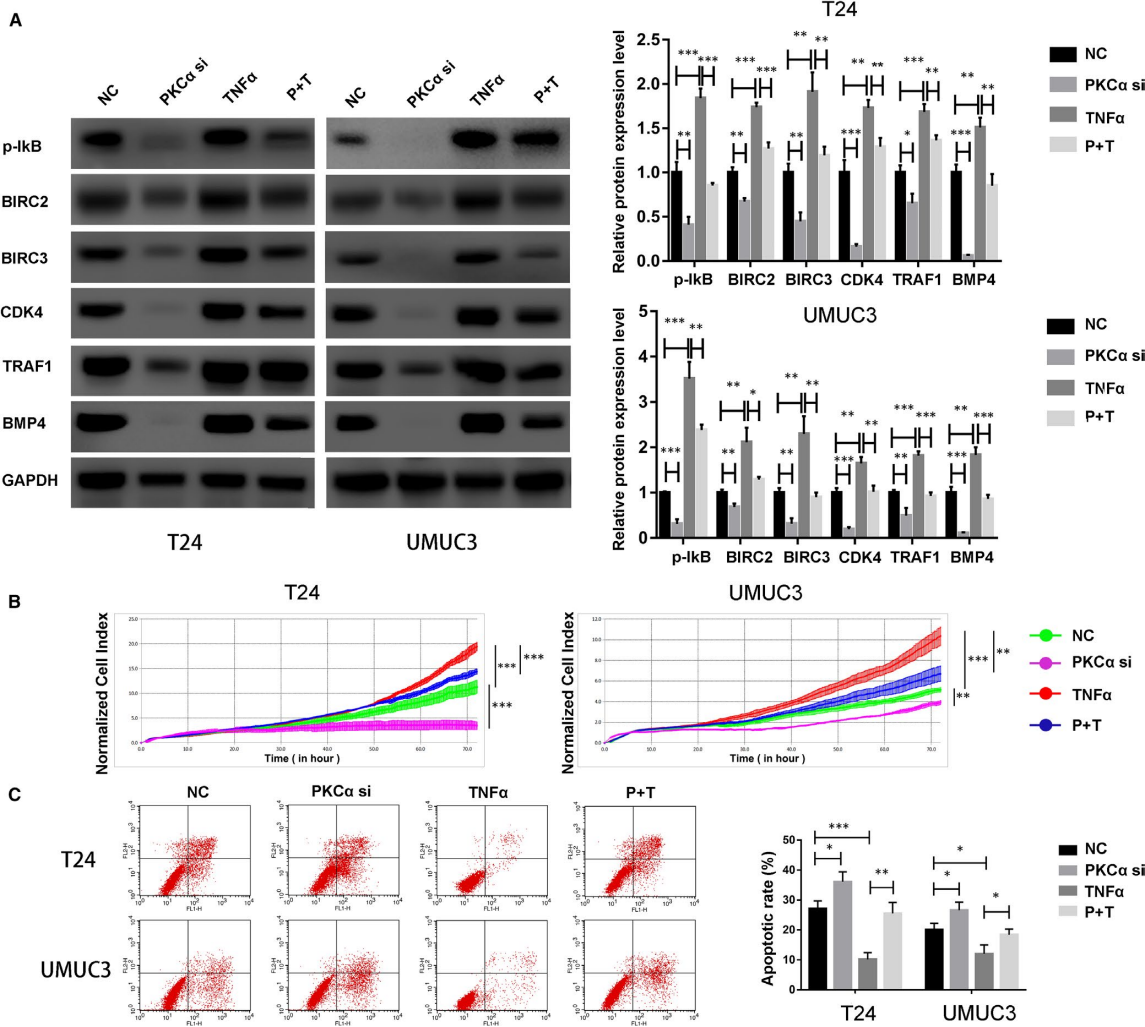
PKCα affects cell proliferation and apoptosis in an NF‐kB–dependent manner in bladder cancer cells. (A) T24 and UMUC3 cells were transfected with PKCα siRNA, TNF‐α (20 ng/mL) or both. The relative expression of p‐IkB and the five target genes (BIRC2, BIRC3, CDK4, TRAF1 and BMP4) was evaluated using Western blot. (B) Proliferation in treated T24 and UMUC3 cells was detected by RTCAs. (C) Apoptosis was estimated in T24 and UMUC3 cells by using flow cytometry

According to GO analysis and KEGG pathway analysis, we found five key genes that were associated with cell proliferation and apoptosis, which participates in the NF‐kB signalling pathway and cancer‐related pathways. To further determine whether the oncogenic role of PKCα in BC depends on the NF‐kB signalling pathway, we transfected cells with PKCα siRNA (PKCα si), TNF‐α (20 ng/mL) or both (P + T). Interestingly, we observed that all five genes were regulated by the PKCα/NF‐kB axis according to Western blotting (Figure 5A). Additionally, we performed the RTCA and flow cytometry and observed that the activation of the NF‐kB signalling pathway significantly promoted proliferation and reduced apoptosis of PKCα‐depleted T24 and UMUC3 cells (Figure 5B‐C).

### 3.6 The five identified genes affect cell function via the PKCα/NF‐kB axis

**FIGURE 6:**
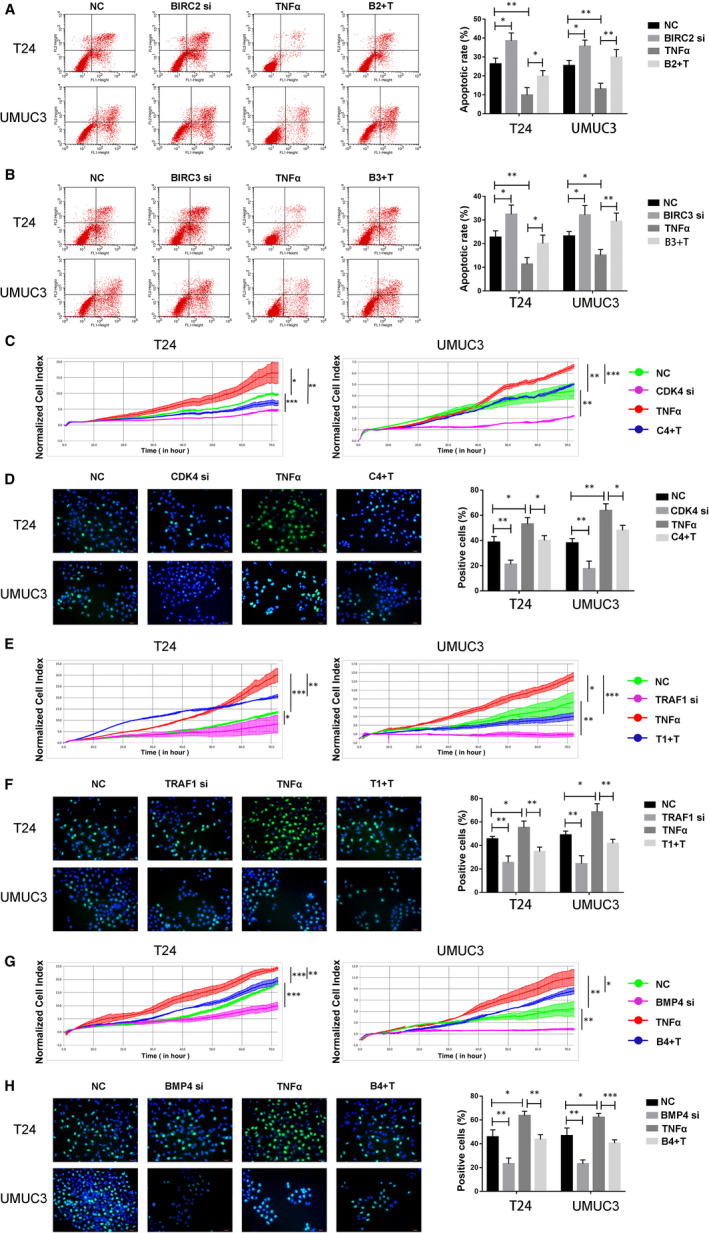
The five key genes affect cell functions regulated by the PKCα/NF‐kB axis. (A and B) T24 and UMUC3 cells were transfected with BIRC2 or BIRC3 siRNA, TNF‐α (20 ng/mL) or both. Apoptosis was estimated in T24 and UMUC3 cells by using flow cytometry. (C to H) T24 and UMUC3 cells were transfected with CDK4, TRAF1 or BMP4 siRNA, TNF‐α (20 ng/mL) or both. Proliferation in treated T24 and UMUC3 cells was detected by EdU or RTCAs

Given the association between the PKCα/NF‐kB axis and both proliferation and apoptosis, we sought to directly test whether the five key genes would affect cell function in a PKCα/NF‐kB–dependent manner. First, we transfected cells with siRNA targeting the five genes (BIRC2, BIRC3, CDK4, TRAF1 and BMP4), TNF‐α (20 ng/mL) or both (B2 + T, B3 + T, C4 + T, T1 + T and B4 + T). Then, proliferation and apoptosis were determined by the RTCA and flow cytometry. As a result, we observed that knockdown of BIRC2 and BIRC3 combined with TNF‐α activation significantly induced apoptosis compared with TNF‐α group in T24 and UMUC3 cells (Figure 6A‐B). Meanwhile, knockdown of CDK4, TRAF1 and BMP4 combined with TNF‐α activation significantly inhibited proliferation compared with TNF‐α group in T24 and UMUC3 cells (Figure 6C‐H).

## 4 DISCUSSION

Though much progress has been achieved in understanding the mechanism of tumorigenesis, many targeted therapies are emerging for several kinds of cancers. Immune checkpoint inhibitors with their favourable toxicity profiles and notable antitumour activity have ushered in a new era in the treatment of advanced urothelial cancer (UC) with five agents targeting the PD‐1/ PD‐L1 pathway being recently approved by the US Food and Drug administration. As BC is characterized by easy recurrence and metastasis, many of the mechanisms leading to BC progression remain unknown. Accumulating evidence has revealed that PKCα is overexpressed in multiple types of cancers, such as breast, renal, lung and colorectal cancer. In addition, PKCα, considered to be an oncogene, regulates the proliferation, invasion, migration and apoptosis of tumour cells in association with many signalling pathways.

Both BIRC2 and BIRC3 are apoptosis‐related proteins and members of the inhibitor of apoptosis (IAP) family; all IAP family members functioning in biological or pathological processes depend on the presence of a baculovirus inhibitor of apoptosis repeat (BIR) domain. Emerging evidence has demonstrated that IAPs are important signalling molecules in many pathological processes, such as inflammation, endoplasmic reticulum stress and some cancers.^9^ Much progress has been made in understanding the mechanism of BIRC2 and BIRC3 in multiple cancers, including liver cancer, chronic lymphocytic leukaemia, breast cancer and ovarian cancer.^10^ In addition to their roles in apoptosis, BIRC2 and BIRC3 participate in several other cellular processes, including proliferation, migration, cell cycle and signal transduction.^11^ However, the mechanism underlying how BIRC2 and BIRC3 promote tumorigenesis remains unexplored. In our study, a moderate positive correlation was observed between the expression of PKCα and that of both BIRC2 and BIRC3. Furthermore, PKCα knockdown could significantly induce BC cell apoptosis through the NF‐kB/BIRC2 or NF‐kB/BIRC3 axes.

CDK4 (cyclin‐dependent kinase 4) is a member of the cyclin‐dependent kinase family comprising serine/threonine kinases. CDK4 was the first kinase considered to be an important regulator of the cell cycle, which could induce G1/S cell cycle transition or G2/M cell cycle transition.^12^ However, emerging evidence has demonstrated that CDK4 is involved in cellular processes other than cell cycle. CDK4 is also essential for regulating proliferation, migration, invasion and apoptosis.^13^ Dysregulation of CDK4 could lead to multiple pathological processes, diseases or even cancer. Many cancers, including breast cancer, acute myeloid leukaemia, non–small‐cell lung cancer and ovarian cancer, have been found to be associated with the dysregulation of CDK4. Accumulating studies revealed that aberrant CDK4 could participate in many cellular pathways associated with malignancies, including the PI3K/Akt signalling pathway in lung cancer, the Wnt/β‐catenin signalling pathway in BC and the JAK‐STAT signalling pathway in gastric cancer.^14^, ^15^, ^16^ Among these, the mechanism by which CDK4 contributes to breast cancer progression has been thoroughly studied, and CDK4 inhibition has been applied to a wide range of human cancers.^17^, ^18^, ^19^ Therefore, developing inhibitors targeting CDK4 and understanding their anticancer effects have garnered increasing interest in recent years.^20^, ^21^ In this study, we found a moderate positive correlation between the expression of PKCα and that of CDK4. Furthermore, PKCα knockdown significantly inhibited BC cell proliferation through the NF‐kB/CDK4 axis.

TRAF1 (tumour necrosis factor receptor–associated factor 1) is a member of the TNF receptor (TNFR) family, which plays significant roles in cell signal transduction, and is considered to be a key adaptor molecule. Some studies indicate that TRAF2 could recruit BIRC2 and BIRC3 to form a protein complex that can facilitate the recruitment of TRAF1.^22^ All these processes contribute to TNF‐induced activation of the TRAF1 signalling pathway. New research has also shown that TRAF1 affects the cell cycle in cancer in a CDK4‐dependent manner. An increasing number of studies revealed that TRAF1 could participate in many cellular pathways associated with pathophysiological processes; these pathways include the P‐38 MAPK, NF‐kB and Akt signalling pathways.^23^, ^24^ Because of its extensive functionality in pathophysiological processes, TRAF1 has been reported to be involved in various diseases, including ulcerative colitis, rheumatoid arthritis, hepatic steatosis and, especially, various cancers.^25^, ^26^ In the present study, we found a moderate positive correlation between the expression of PKCα and that of TRAF1. Furthermore, PKCα knockdown significantly inhibited BC cell proliferation through the NF‐kB/TRAF1 axis.

BMP4 (bone morphogenetic protein‐4) belongs to the BMP family, which was originally considered to comprise proteins that induce new bone formation. The BMP family was eventually reported to be a member of the transforming growth factor b (TGF‐b) superfamily, whose members play key roles in signal transduction.^27^ In addition to its significant role in skeletogenesis, BMP4 was revealed to be an important regulator in many diseases, such as Alzheimer's disease, pathological cardiac hypertrophy/heart failure, diabetic nephropathy and multiple cancers. BMP4 has emerged in many cancers, including breast cancer, prostate cancer, colorectal cancer and non–small‐cell lung cancer.^28^ In some cancers, BMP4 was also reported to be associated with poor prognosis.^29^, ^30^ In the present study, we found a moderate positive correlation between the expression of PKCα and that of BMP4. Furthermore, PKCα knockdown significantly inhibited BC cell proliferation through the NF‐kB/BMP4 pathway.

First, our findings revealed that PKCα knockdown inhibited cell proliferation, invasion and migration, promoted apoptosis and induced G1/S cell cycle arrest in BC cells in vitro, whereas PKCα up‐regulation resulted in the opposite effects in vivo. According to the genome‐wide microarray analysis, knockdown of PKCα led to the up‐regulation or down‐regulation of multiple downstream target genes. Then, we used bioinformatic analysis to detect a correlation between PKCα and cellular processes, including cell proliferation, migration, invasion and apoptosis. Finally, we found five key downstream genes, BIRC2, BIRC3, CDK4, TRAF1 and BMP4 that were associated with PKCα. All five target genes were positively regulated by PKCα, and all five genes showed a moderate positive correlation with the expression of PKCα. BIRC2 and BIRC3 were associated with apoptosis, and CDK4, TRAF1 and BMP4 were associated with proliferation. In addition, all these five key genes participated in proliferation, and apoptosis was regulated by the PKCα/NF‐kB axis. In particular, although PKCα does not regulate the cell cycle of bladder cancer through the NF‐kB signalling pathway, previous studies have found that PKCα can regulate the expression of UNC5B, and UNC5B can regulate the cell cycle of bladder cancer. Therefore, we suggested that PKCα may regulate the cell cycle of bladder cancer by regulating UNC5B. But this needs our later experimental verification. In this article, we verify the function of PKCα and find out the signalling pathways and related factors that may affect it through chip analysis, and assume their relevance, we finally through a series of experiments to verify the PKCa through regulating NF‐kB signalling pathway, in turn, affect the five key factor, ultimately affect the function of bladder cancer cell biology. All these data may serve as a basis for the development of new clinical diagnostic and prognostic markers and provide new insight into the treatment of BC.

## CONFLICT OF INTEREST

All authors give consent for the publication of the manuscript. The authors declare no competing financial interests. All authors read and approved the final manuscript.

## AUTHOR CONTRIBUTIONS

**Xiaotong Zhang:** Investigation (equal); Writing‐original draft (equal); Writing‐review & editing (equal). **Jiarun Zhang:** Investigation (equal). **Hao Zhang:** Software (equal). **Yang Liu:** Formal analysis (equal). **Lei Yin:** Investigation (equal). **Xi Liu:** Visualization (equal). **Xuejie Li:** Writing‐original draft (equal). **Xiuyue Yu:** Methodology (equal). **Jinlong Yao:** Investigation (equal). **Zhe Zhang:** Funding acquisition (equal). **Chuize Kong:** Funding acquisition (equal); Project administration (equal).

## Data Availability

The data sets used and analysed during the current study are available from the corresponding author upon reasonable request.
